# Adeno-associated viral (AAV) vector-mediated therapeutics for diabetic cardiomyopathy – current and future perspectives

**DOI:** 10.1042/CS20210052

**Published:** 2021-06-02

**Authors:** Darnel Prakoso, Mitchel Tate, Miles J. De Blasio, Rebecca H. Ritchie

**Affiliations:** 1 Departments of Drug Discovery Biology, Monash Institute of Pharmaceutical Sciences, Monash University Parkville Campus, Australia; 2 Diabetes, Monash University, Clayton, Victoria 3800, Australia; 3 Pharmacology, Monash University, Clayton, Victoria 3800, Australia

**Keywords:** AAV, diabetes, Diabetic Cadiomyopathy, Gene Therapy

## Abstract

Diabetes increases the prevalence of heart failure by 6–8-fold, independent of other comorbidities such as hypertension and coronary artery disease, a phenomenon termed diabetic cardiomyopathy. Several key signalling pathways have been identified that drive the pathological changes associated with diabetes-induced heart failure. This has led to the development of multiple pharmacological agents that are currently available for clinical use. While fairly effective at delaying disease progression, these treatments do not reverse the cardiac damage associated with diabetes. One potential alternative avenue for targeting diabetes-induced heart failure is the use of adeno-associated viral vector (AAV) gene therapy, which has shown great versatility in a multitude of disease settings. AAV gene therapy has the potential to target specific cells or tissues, has a low host immune response and has the possibility to represent a lifelong cure, not possible with current conventional pharmacotherapies. In this review, we will assess the therapeutic potential of AAV gene therapy as a treatment for diabetic cardiomyopathy.

## Diabetic cardiomyopathy

Diabetes is a major health problem worldwide, responsible for approximately 5 million deaths in 2017, with an increasing incidence expected to reach 693 million by the year 2045 [1]. One of the leading causes of death (approximately 50%) in people with diabetes is cardiovascular disease. In particular, the prevalence of heart failure in diabetic patients is reportedly increased by 6–8-fold in the 45- to 65-year-old age-group compared with non-diabetic individuals [2]. Moreover, diabetes alone can accelerate the development of heart failure in individuals with pre-existing cardiac pathologies (such as myocardial infarction), resulting in poorer prognosis compared with non-diabetic individuals [3]. The accompanying abnormalities in cardiac structure and function are collectively termed ‘diabetic cardiomyopathy’ or diabetes-associated heart failure, originally defined as cardiomyopathy not directly attributable to hypertension or coronary disease. This phenomenon has been known for at least 5-decades [4–7]; however, diabetic cardiomyopathy still remains the subject of intense research to understand the key causal signalling pathways to target for future therapeutic development.

### Key signalling pathways implicated in diabetes-induced cardiac dysfunction and remodelling

**Figure 1:**
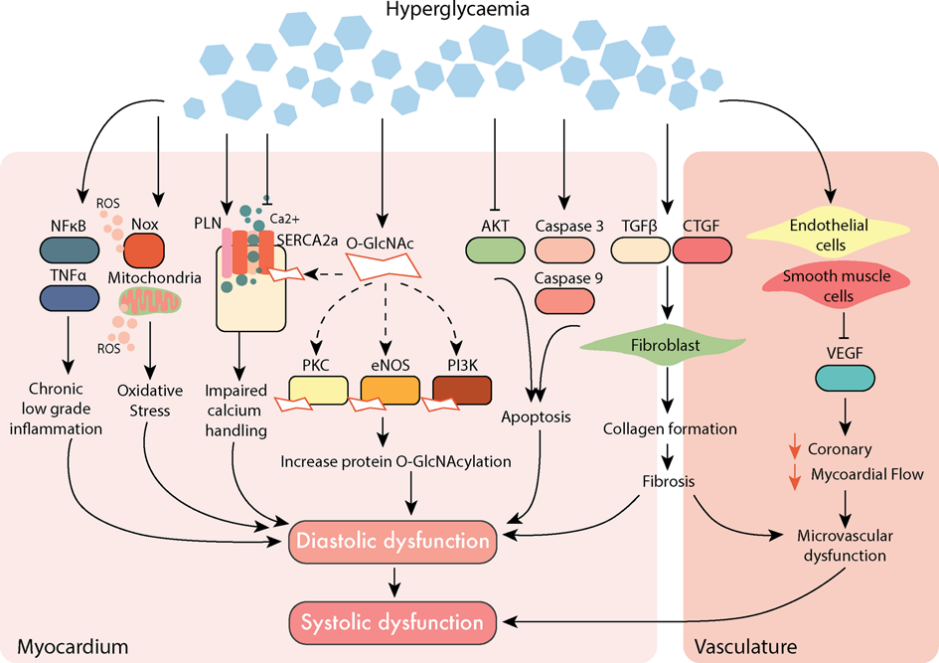
Overview of dysregulated major signalling pathways dysregulated in diabetic cardiomyopathy Diabetes-induced molecular impairments lead to cardiac remodelling, inflammation, oxidative stress and impaired calcium handling, contributing to cardiac dysfunction that initially develops as diastolic dysfunction. Over time, impairment of endothelial and smooth muscle cells in the vasculature can lead to reduced coronary microvascular blood flow, as a result of this microvascular dysfunction. The dysfunction in the vasculature and the myocardium can eventually lead to LV systolic dysfunction; Akt, protein kinase B; CTGF, connective tissue growth factor; eNOS, endothelial nitric oxide synthase; HBP, hexosamine biosynthesis pathway; LV, left ventricular; NFκB, nuclear factor-κB; Nox, NADPH oxidase; PI3K, phosphoinositide-3 kinase; PKC, protein kinase-C; PLN, phospholamban; ROS, reactive oxygen species; SERCA2a, sarcoplasmic/endoplasmic reticulum ATPase- Ca^2+^; TGF-β, transforming growth factor-β; TNFα, tumour necrosis factor-α (see text for references).

To date, several contributing mechanisms have been implicated in the development of diabetic cardiomyopathy, each of which has an impact on cardiac function and pathological cardiac remodelling (summarised in Figure 1). For instance, long-term hyperglycaemia is associated with chronic systemic low-grade inflammation. Impaired glucose handling leads to the activation of pro-inflammatory factors such as nuclear-factor-κB (NF-κB) and tumour necrosis factor-α (TNF-α) that have been linked to insulin resistance and an array of pathological consequences of diabetes [8,9]. This is paired with excessive reactive oxygen species (ROS) production in the myocardium, via the dedicated ROS-producing enzyme, NADPH oxidase (Nox), and as a result of mitochondrial electron transport chain uncoupling [7]. Excessive ROS, their dysregulation and/or impaired antioxidant defences result in oxidative stress, a key hallmark observed in the diabetic heart [10]. Diabetes-induced impairments in Ca^2+^ handling can impact cardiomyocyte function, prolonging relaxation and impairing contractile function [11]. In preclinical animal models of both type-1 and type-2 diabetes, a reduction in the expression and activity of the Ca^2+^ handling protein, sarcoplasmic/endoplasmic reticulum Ca^2+^-ATPase (SERCA2a), and/or an increase in its regulator, phospholamban (Pln), impairs cardiac function [12]. Likewise, these Ca^2+^ handling proteins have been shown to undergo post-translational modification in the setting of hyperglycaemia, altering their cellular function. O-GlcNAcylation is one such example, where a rapid and dynamic attachment of the sugar moiety, O-GlcNAc, to nuclear and cytoplasmic proteins at serine (Ser) or threonine (Thr) residues, results in altered function of a broad range of proteins [13]. In the cardiovascular system, SERCA2a, protein-kinase C, endothelial nitric oxide synthase (eNOS) and phosphoinositide-3 kinase (PI3K) are all O-GlcNAc modified [14]. For example, in mice with diabetic cardiomyopathy, left ventricular (LV) diastolic dysfunction develops in conjunction with elevated levels of global cardiac protein O-GlcNAcylation [15]. This occurs in conjunction with the inhibition of pro-survival kinase Akt, leading to an increase in apoptosis in the diabetic heart [16]. Although programmed cell death rarely occurs in the healthy myocardium (as cardiomyocytes rarely proliferate in adult cardiac muscle), it is a feature of the diabetic heart and end-stage heart failure [17]. This is driven by diabetes-induced activation of cell death factors, including caspase-3 and caspase-9 [18]. Over time, excessive cell death can cause tissue damage and scar formation. The deposition of extracellular matrix consists predominantly of collagen produced by activated fibroblasts cells (known as myofibroblasts) that stimulate transforming growth factor-β (TGF-β) and connective tissue growth factor (CTGF) to activate cell surface receptors and their response to cytokines [19,20]. Maladaptive remodelling eventually leads to a clinically observable phenotype, characterised by cardiac fibrosis and LV diastolic dysfunction that eventually leads to systolic dysfunction [21]. In addition to the direct cardiac effects, diabetes can cause microvascular dysfunction via impairment in endothelial and smooth muscles cells, impacting coronary and myocardial blood flow [7]. This vascular dysfunction may often not be evident until later in the disease progression, with initial inflammatory infiltration of the microvascular that lead to vascular remodelling and perivascular fibrosis [22]. This further contributes to increased thickness of the arterioles with a reduction in the number of capillaries and arterioles [23]. Similarly, pre-clinical models of type-1 diabetes have been demonstrated to have a similar phenotype, in addition to the reduction of important angiogenic growth factors such as the vascular endothelial growth factor (VEGF) [24]. These signalling responses contribute to the overall pathophysiology of diabetic cardiomyopathy and have been comprehensively reviewed recently [7].

### Current treatments for the diabetic heart

Although the pathophysiology of diabetic cardiomyopathy is well-established, effective treatment options approved for clinical use are limited. Early studies indicated that lower glycated haemoglobin (a surrogate measure of long-term blood glucose) is associated with a lower incidence of cardiovascular disease in diabetic patients [25]. However, when this is applied to high-risk diabetic patients, the opposite occurs; it worsens the outcome of cardiovascular events [26]. Thus, lowering blood glucose alone is often not sufficient to prevent the development of diabetic cardiomyopathy.

Currently in the clinic, several different pharmacological agents are routinely used to manage diabetic cardiomyopathy. First-line treatments include those targeting glucose control as well as those targeting cardiovascular pathology. This includes for example renin–angiotensin system inhibitors (angiotensin converting-enzyme inhibitors, ACEi, and angiotensin receptor blockers) and metformin, which have been shown to be safe and to delay cardiac disease progression in diabetic patients, at least to some extent [27,28]. Thiazolidinediones (TZDs), a class of drug effective in lowering blood glucose levels, have been shown clinically to increase the incidence of hospitalisation due to heart failure, thus rendering them unsuitable as a treatment for diabetic cardiomyopathy [29]. More recently, treatments that modulate blood glucose levels via the incretin system have shown some efficacy in terms of cardiovascular outcomes. Although the incretin system potentiators, dipeptidyl peptidase-4 (DPP4) inhibitors, are associated with a neutral or increased risk of developing heart failure in the clinic, new-generation incretin system mimetics (glucagon-like peptide-1 (GLP-1) receptor agonists) have demonstrated favourable cardiovascular outcomes with a good safety profile [30,31]. Sodium-glucose co-transporter-2 (SGLT-2) inhibitors, initially indicated specifically for the management of type-2 diabetes, have demonstrated a marked beneficial effect in terms of cardiovascular complications in the setting of diabetes [32,33]. The beneficial effects of SGLT2 inhibitors were observed as a result of the now mandated requirement for clinical investigation of cardiovascular outcomes in diabetic drugs [34]. Whilst SGLT2 inhibitors reduce cardiovascular risk, mechanistic insights into how they impact heart failure in T2D is lacking [7,35]. Further, one recent clinical trial on SGLT-2 inhibitors suggests these beneficial effects may extend to patients with heart failure irrespective of diabetes, suggesting a mechanism independent of blood glucose-lowering [36]. For a small number of patients, risk of adverse outcomes (e.g. increased risk of amputation, genital infections or worsening kidney function) may preclude their suitability [37,38]. For the majority of diabetic patients, these newer glucose-lowering agents have proven to be favourable for limiting the risk of heart failure hospitalisation. There remain some patients however who will still require alternative therapeutic options to address the underlying triggers of, or reverse the cardiac damage associated with, diabetes. As the body of evidence continues to grow with respect to the treatment of diabetic cardiomyopathy (whether via SGLT2 inhibition or other approaches), this will also influence the need for more targeted approach for future interventions. One such potential avenue of treatment, which may also provide a longer-term treatment solution, is the use of adeno-associated viral vector-mediated (AAV) gene therapy.

## AAV-based therapeutics represent a new frontier in clinical medicine

Treatment options for cardiovascular diseases have advanced significantly in recent years, as a result of improved understanding of the molecular pathways involved in cardiac damage [39,40]. In particular, the use of gene therapy has proven to be one of the most promising avenues to treat various types of diseases, including heart failure [40]. Aimed at the correction of key pathologies, gene therapy involves the delivery of therapeutic genes of interest to a specific tissue target. Successful incorporation of the delivered gene allows the endogenous cell machinery to produce the specific encoded protein [41,42]. The success of gene therapy depends heavily on the efficient transfer of the genetic material to the tissue of interest, which is facilitated by different delivery strategies. Early delivery methods included the use of naked plasmid DNA, liposomal DNA complexes, polymer-carrying DNA and oligonucleotides [43]. Delivery of naked plasmids to tissues, however, does not provide sufficient transfection into the tissue of interest [44,45]. Furthermore, rapid systemic degradation of plasmids and poor cellular entry are other limitations [40,46,47]. In contrast, non-pathogenic viral approaches have been shown to be a more superior method as the vector for gene delivery [45,46]. Notably, AAVs have been demonstrated to exhibit the best risk-benefit profile and will be the focus for the remainder of this review.

### Brief introduction to AAV principles

**Figure 2:**
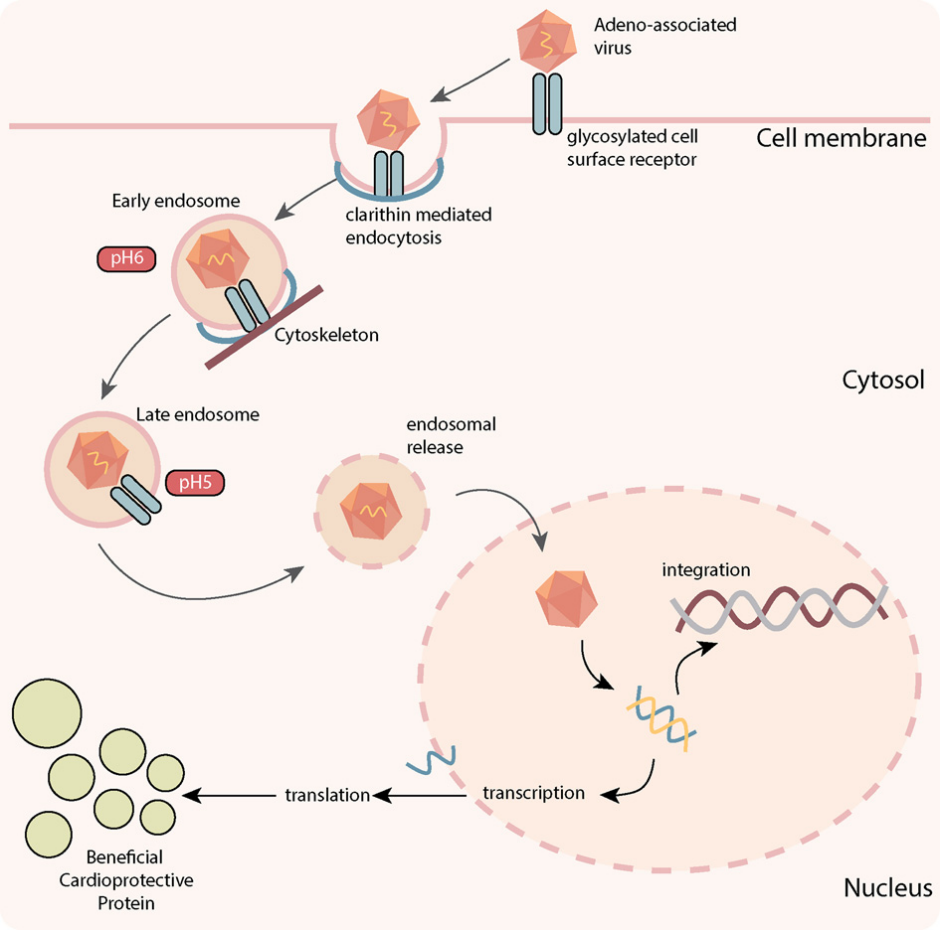
Principles of AAV-mediated gene therapy Adeno-associated virus (AAV) binds to the host glycosylated cell surface receptor to trigger clathrin-mediated endocytosis internalization. The AAV then moves through the cytosol via the cytoskeletal network. Conformational changes are then triggered by pH changes in the cellular environment, leading to endosomal release. The AAV undergoes transport to the nucleus, releasing its cargo, where it is then transcribed into double-stranded DNA for transcription, or undergoes integration to the host genome (which rarely occurs). Messenger RNA produced from transcription of the cargo leads to its translation to the protein-of-interest outside of the nucleus. Production of this protein-of-interest then enables the cardioprotective effects that are observed in response to AAV-mediated gene therapy.

Derived from the *Parvoviridae* family, AAVs are non-enveloped single-stranded DNA vectors, with a favourable safety profile and the capability of achieving persistent transgene expression in a wide range of target tissues, including cardiac tissue [48]. AAVs are relatively small (20 nm) and therefore limited in their packaging capacity of only around 4.7 kb [47]. Yet one of the most attractive features of AAV vectors is the continued expression of the transgene for a prolonged period of time [40,49], despite the extrachromosomal location of the vector [47]. However, the infrequent integration of the vector means that transduction must occur in cells that either do not replicate or do so very slowly [47]. Cardiomyocytes are an excellent example of cells that are considered the most compatible for use of AAV gene therapy, as cardiomyocyte turnover is negligible in adults [50]. For *in vivo* gene delivery, recombinant AAVs (rAAV) are commonly used, which have the same sequence and structure as a wild-type AAV, but are devoid of all AAV-protein coding sequences [51]. AAVs enter the cell via glycosylated cell surface receptors, triggering clathrin-mediated endocytosis (Figure 2) [51]. Utilising the cytoskeletal network AAVs advance through the cytosol, undergoing conformational modification in response to a change in pH [51]. AAVs are then released by the endosome, where they enter the nucleus and release their content. The viral inverted terminal repeats (ITRs) present in the rAAV genome can drive inter- or intra-molecular recombination to form circularised episomal genomes that can persist in the nucleus. Vector genomes can also undergo integration into the host genome at very low frequencies; however, this is a very rare occurrence unlike both lentiviral and retroviral that can randomly integrate into the host genome to disrupt normal gene function [52]. Moreover, compared with adenoviruses which were a popular vector choice in the early 2000s, AAVs has been shown to be excellent to evade the innate immune system [53] and considered highly safe and potent compared with other vectors.

### AAV design for cardiac-targeted transduction

To achieve efficient cardiac transduction, multiple factors must be considered, including the identification of naturally cardiotropic AAVs. Currently, more than 100 serotypes of wild-type AAVs have been reported, each with distinct tissue tropism, as determined by their capsid protein structures [41,54]. Among these serotypes, AAV1, AAV6, AAV8 and AAV9 have been identified as the most cardiotropic serotypes for systemic delivery [55]. It is also important to note that these serotypes target other organs, including the liver for AAV9 and both the lung and skeletal muscle for AAV6 [55]. These have led to various efforts that include the generation of chimeric AAVs by shuffling from various subtypes to improve transduction efficacy and transgene expression [56].

In addition to identifying the most effective cardiotropic/organ-specific serotype, promoter selection is another major element to consider. The combination of both allow the mediation of AAV expression and enable the control of gene transcription that is delivered [57]. Promoters are usually located upstream of the gene of interest and are 100–1000 base pairs in length. This can either stimulate or repress transcription initiation at the transcription start site, mediated by the RNA polymerase II in the core promoter. One of the most commonly used promoters, cytomegalovirus (CMV), can achieve both strong and robust transgene expression [58]. Inducible promoters such as the tetracycline and doxycycline systems have also been used to manipulate transgene expression in preclinical studies [59,60]. For cardiac transduction, cardiac-specific promoters such as α-myosin heavy chain (αMHC), troponin and myosin light chain (MLC) have been commonly used to drive expression throughout the heart, including in the ventricles and atria [61–64]. Interestingly, the combination of AAV6 (which has strong skeletal muscle tropism) and a CMV promoter favours cardiac muscle transduction, through a mechanism that is not well understood [65]. More recently, atrial natriuretic factor (ANF) has been suggested to confer atrial-specific transduction, without observable transduction in the ventricles [66]. Therefore, promotor selection is an important consideration in AAV design, where further research will likely improve our ability to limit expression to individual organs, compartments or even cell types.

## AAV-based therapies for diabetic cardiomyopathy

The increased use of AAV gene therapy in the biomedical field has led to an intensified effort to identify novel potential treatments for diabetic cardiomyopathy. This can be done by targeting the underlying mechanism of the cardiac pathology, not possible using conventional pharmacotherapies. Below we discuss recent findings that aim to treat diabetic cardiomyopathy and other cardiovascular diseases (Table 1).

**Table 1:** List of gene therapies investigated in preclinical and clinical studies

Target	Mechanism of action	Vector serotype	Dose (vector genomes)	Study phase	Model	Indication	Delivery method	References
Preclinical studies								
S100A1	Increased calcium handling protein	rAAV6	2.5 × 10^11^ vg	Small animal	Rodents	Ischaemic heart failure (myocardial infarction)	Coronary perfusion	[69]
		AAV9	1.5 × 10^13^ vg	Large animal	Porcine	Ischaemic heart failure (myocardial infarction)	Retrograde coronary injection	[70]
		AAV6	1.5 × 10^13^ vg	Large animal	Porcine	Ischaemic heart failure (myocardial infarction	Retrograde coronary injection	[71]
SUMO-1	Increased calcium handling protein	rAAV9	5 × 10^10^ vg	Small animal	Rodents	Heart failure transverse aortic constriction	Tail vein injection	[72]
		AAV1	5 × 10^12^ vg 1 × 10^13^ vg	Large animal	Porcine	Ischaemic heart failure (myocardial infarction)	Antegrade intracoronary infusion	[73]
I-1	Increased calcium handling protein	AAV9	2.8×10^12^ vg	Small animal	Rodents	Heart failure transverse aortic constriction	Tail vein injection	[75]
		BNP116^1^	3 × 10^12^ vg 1 × 10^13^ vg	Large animal	Porcine	Ischaemic heart failure (myocardial infarction)	Intracoronary infusion	[74]
		BNP116^1^	1 × 10^13^ vg 1 × 10^14^ vg	Large animal	Porcine	Non-ischaemic HF (volume overload HF)	Intracoronary injection	[76]
Urocortin	Increase calcium protein handling	AAV8	5 × 10^11^ vg	Small animal	Rodents	Ischaemic heart failure (myocardial infarction)	Jugular vein injection	[78]
		AAV8	5×10^11^ vg	Small animal	Rodents	Cryoinjury myocardial infarction model	Jugular vein injection	[79]
VEGF-B	Increased angiogenesis	AAV9	1 × 10^10^ vg	Small animal	Rodent	Heart failure transverse aortic constriction	Direct myocardial injection	[81]
		AAV9	1 × 10^13^ vg 2 × 10^13^ vg 5 × 10^13^ vg	Large animal	Canine	Non-ischaemic dilated cardiomyopathy	Intracoronary infusion	[82]
NGF	Increased angiogenesis	AAV2^2^ AAV9^3^	1 × 10^11^ vg 1.5 × 10^12^ vg	Small animal	Rodents	Diabetic cardiomyopathy	Direct myocardial injection^2^ and tail vein injection^3^	[86]
YAP	Cardiac regeneration	AAV9	N/A	Small animal	Rodents	Myocardial infarction	Direct myocardial injection	[87]
FGF-2 sTGFβ2	Growth modulators	AAV8	1 × 10^10^ vg 1 × 10^11^ vg	Small animal	Rodents	Heart failure ascending aortic constriction	Retro orbital injection	[88]
SOD	Increase antioxidant defence	AAV	2.5 × 10^10^ vg 5 × 10^10^ vg 2.5 × 10^12^ vg	Small animal	Rodents	Ischaemia reperfusion injury	Direct myocardial injection	[90]
CTRP3	Limit ROS and inflammation	AAV	5×10^11^ vg	Small animal	Rodents	Diabetic Cardiomyopathy	Tail vein injection	[91]
HO-1	ROS scavenger and anti-inflammatory	AAV2	2×10^11^ vg	Small animal	Rodents	Ischaemic heart failure (myocardial infarction)	Direct myocardial injection	[92]
		AAV6	1 × 10^13^ vg	Large animal	Porcine	Ischaemic heart failure (myocardial infarction)	Retro infusion into the ventricular vein	[93]
BFIB4	Increased longevity factors and anti-inflammatory	AAV9	1 × 10^12^ vg	Small animal	Rodents	Diabetic cardiomyopathy	Tail vein injection	[94]
RNR	Increase pro-survival protein (via increased energy synthesis)	rAAV6	2.5 × 10^13^ vrg	Small animal	Rodents	Ischaemic heart failure (myocardial infarction)	i.v. injection via retro-orbital sinus route	[96]
		AAV6	1 × 10^12^ vrg 5 × 10^12^ vrg 1 × 10^13^ vrg	Large animal	Porcine	Ischaemic heart failure (myocardial infarction)	Antegrade Intracoronary infusion	[97]
βARKct	Inhibition of β-adrenergic	AAV6	1 × 10^13^ vg	Large animal	Porcine	Ischaemic heart failure (myocardial infarction)	Retrograde injection into coronary veins	[99]
O-GlcNAcylation	Alteration of cardiac O-GlcNAc balance	AAV6	2 × 10^11^ vg 1 × 10^12^ vg	Small animal	Rodents	Diabetic cardiomyopathy	Tail vein injection	[103,104]
PIM-1	Increased pro-survival kinase	AAV9	1 × 10^10^ vg 5 × 10^10^ vg	Small animal	Rodents	Diabetic cardiomyopathy	Tail vein injection	[106]
PI3K(p110α)	Increase pro-survival kinase and reduce ROS	AAV6	2 × 10^11^ vg	Small animal	Rodents	Diabetic cardiomyopathy	Tail vein injection	[108,109]
miRNA-1	mRNA regulator	AAV9	5 × 10^11^ vg	Small animal	Rodents	Heart failure ascending aortic constriction	Tail vein injection	[111]
miRNA-21	mRNA regulator through gelsolin inhibition	AAV9	N/A	Small animal	Rodents	Diabetic cardiomyopathy	Tail vein injection	[112]
miRNA-30c	mRNA regulator to increase PPARα	AAV9	1 × 10^11^ vg	Small animal	Rodents	Diabetic cardiomyopathy	Tail vein injection	[113]
miRNA-320	mRNA regulator to increase CD36 expression	AAV9	1 × 10^11^ vg	Small animal	Rodents	Diabetic cardiomyopathy	N/A	[114]
Clinical Studies								
SERCA2a	Increased calcium handling protein	AAV1	1 × 10^13^ vg	Phase 2b	Human	Heart failure	Intracoronary infusion	[117]
Adenylyl Cyclase 6	Increased calcium handling and pro-survival kinase	Adv	3.2 × 10^9^ vg 3.2 × 10^10^ vg 1 × 10^11^ vg 3.2 × 10^11^ vg 1 × 10^12^ vg	Phase 2b	Human	Heart failure	Intracoronary injection	[118]

Summary of studies that have used AAV gene therapy to target different types of heart failure in preclinical and clinical studies. Vectors: ^1^Chimeric AAV between AAV2 and AAV8 ^ 2^First intervention ^3^Second intervention. AAV, adeno-associated viral; Adv, adenoviral; βARKct; β-adrenergic receptor kinase 1 (carboxy terminus); BFIB, bactericidal/permeability-increasing fold-containing family B member 4; CTRP3, C1q/tumour necrosis factor-related protein; FGF, fibroblast growth factor-2; HO-1, heme oxygenase-1; miRNA, micro RNA; NGF, nerve growth factor; PI3K(p110α), phosphoinositide 3- kinase (p110α); PIM-1, pro-viral integration site for Moloney murine leukaemia virus; RNR, ribonucleotide reductase;S100A1, S100 calcium-binding protein A1; SERCA2a; sarcoplasmic/endoplasmic reticulum ATPase-2SOD, superoxide dismutase; sTGFβ2, soluble transforming growth factor- β2; SUMO-1, small ubiquitin-related modifier-1; I-1, constitutively active inhibitor-1; VEGF-B, vascular endothelial growth factor B; YAP, yes-associated protein 1.

### Calcium cycling/handling-targeted AAVs

The S100 calcium-binding protein A1 (S100A1) is involved in all Ca^2+^-dependent target protein interactions and has recently been discovered to play a critical role in heart failure [67]. S100A1 acts upstream of SERCA2a to modulate a wide range of cellular effects. The protein interacts with Pln to increase the activity of SERCA2a and regulates ryanodine receptor-2 (Ryr2) function, during both systole and diastole [68]. In the setting of heart failure (where S100A1 is down-regulated), its restoration via rAAV6-S100A1 in rats restored cardiac function and improve Ca^2+^ handling via a SERCA2a-dependent mechanism [69]. Similar improvements were also observed in large animal models of myocardial infarction that was administered with rAAV9-S100A1 [60]. Moreover, administration of S100A1 AAV has also been proven to be safe as demonstrated in a study conducted in pigs [71]. Equally, another SERCA2a modulator, small ubiquitin-related modifier-1 (SUMO-1) stabilised cellular activity through post-translational modification of both mouse and human proteins [72]. SUMO-1 binds to a broad range of proteins at their lysine residues, resulting in SUMOylation of the protein. In the setting of heart failure (where levels of both SUMO-1 and SUMOylation are diminished), administration of AAV9-SUMO1 in mice increases survival rate and improved cardiac function [72]. This has been confirmed in a porcine model of heart failure, where administration of AAV1-SUMO1 increased specificity protein-1 (Sp-1) SUMOylation, improving cardiac function and stabilisation of LV volumes [73]. In a separate study, another upstream regulator of SERCA2a, inhibitor-1 (I-1), was demonstrated to be cardioprotective in a porcine model of heart failure [64]. I-1 reduced the activity of protein phosphatase-1, an upstream regulator of the SERCA2a-Pln complex and has been shown to decline with heart failure [74,75]. Administration of constitutively active (I-1c)-AAV to both ischaemic and non-ischaemic heart failure in pigs improved cardiac function (systolic and diastolic), in conjunction with increased Pln phosphorylation, leading to enhanced SERCA2a activity [64,65]. In addition to cardiac targeted gene therapy, administration of liver targeted AAV8-Urocortin2 or Urocortin-3 improved cardiac function in the failing murine heart [77]. The approach is unique, as the product of the liver targeted gene therapy does not directly produce the beneficial effects, but rather through the activation of corticotropin-releasing hormone receptor 2 (CRH2) by Urocortin. The activation of CRH2 exerts extensive cardioprotective activity, including increasing calcium handling proteins such as SERCA2a and troponin in the heart [78,79]. In addition to its cardioprotective effects, Urocortin-2 (but not Urocortin-3) gene transfer reduces blood glucose levels and increases glucose clearance, indicating its potential as an ideal candidate for the treatment of diabetic cardiomyopathy [77]. Thus, these studies demonstrate the central role of SERCA2a in maintaining cardiac contractility suggesting its suitability as a therapeutic target for heart failure or diabetic cardiomyopathy. However, direct targeting of other calcium handling approaches using AAV (such as Ryr2 and Pln) should also be considered as a potential therapeutic target to improve cardiac function.

### Growth factor-targeted AAVs

Gene therapeutic approaches targeting growth factors, particularly to induce new blood vessel formation, has been previously explored to treat several cardiovascular diseases. The VEGF family, consisting of VEGF-A, -B, -C, -D and -E, are among the most powerful regulators of blood vessel growth [80]. In particular, VEGF-B has been associated with enhancing cardiac angiogenesis with specific effects on metabolism, cell survival and apoptosis [80]. Previous reports demonstrated that administration of cardiac-specific AAV9-VEGF-B in an aortic constriction mouse model modulated the angiogenic response, increasing proliferation and thereby attenuating systolic function [81]. These findings were corroborated in a canine model of dilated cardiomyopathy, where both LV diastolic and systolic function were preserved, in addition to increases in cardiomyocyte antioxidant defence [82]. Nerve growth factor (NGF) is another growth modulator which is implicated in the promotion of angiogenesis, similar to VEGF-B [83]. Secreted by glycoproteins, NGF elicits its biological effects mainly by binding to high-affinity tropomyosin-related receptor A, leading to inactivation of the forkhead box-O-transcription factor (Foxo) pathway [84]. Overexpression of NGF using gene therapy in mice after myocardial infarction increased both cell survival and cardiac perfusion [85]. Likewise, systemic delivery of human AAV9-NGF prevented cardiomyopathy in diabetic mice, while limiting LV diastolic dysfunction [86]. Similarly, Yes-associated protein (YAP), which has a vital role in regulating embryonic cardiomyocyte proliferation, has attracted interest as a potential mechanism to enhance heart regeneration. By introducing AAV9 carrying a human YAP sequence, contractile function and cell survival were enhanced in mice following myocardial infarction [87]. More recently, a therapeutic gene approach combining two genes (fibroblast growth factor-2 (FGF-2) and soluble transforming growth factor-β-2 (sTGFβ2)) simultaneously in an AAV8 vector limits both obesity and type-2 diabetes phenotypes, and has separately been shown to improve cardiac contraction induced by aortic constriction [88]. These reports highlight the major advantage of utilising AAV to deliver growth factors and modulators to the heart where there is a lack of cell regeneration and replication. This is especially important as off-target or systemic delivery can lead to uncontrollable cancerous growth in other unwanted tissues. Thus, the application of pin-point growth factor delivery using AAV may also favour other organ diseases that require enhance angiogenesis.

### AAVs targeting oxidative stress and inflammation pathways

Increased production of ROS has been shown to cause cellular damage and contribute to the pathology of diabetic cardiomyopathy. One way to limit this is to target superoxide dismutase (SOD), a family of metalloenzymes that scavenge superoxide radicals and convert them to oxygen and hydrogen peroxide. Early studies indicate that the administration of the SOD gene via adenovirus protects from myocardial infarction [89]. A subsequent study revealed a similar outcome with the administration of rAAV-Ec-SOD, where it protected against damage associated with ischaemic–reperfusion injury such as cardiac infarction and dysfunction [90]. However, more recent efforts seem to target the increase in ROS further upstream, leading to the modulation of inflammatory pathways. For instance, administration of rAAV-C1q/tumour necrosis factor-related proteins (CTRPs), a key metabolic regulator of the diabetic heart, demonstrated reduced ROS-producing enzyme activity (Nox and p67 phox) and increase SOD, while also attenuating cardiac inflammation (via reduced TNF-α) [91]. Similarly, targeting the enzyme, heme oxygenase-1 (HO-1), which catalyses the degradation of heme-producing biliverdin (ROS scavenger) and carbon monoxide, has been demonstrated to limit inflammation [92]. Indeed, the administration of rAAV6-human-HO-1 to pigs prior to myocardial ischaemia reperfusion demonstrates reductions in both infarct size and extent of LV systolic dysfunction compared to the control group [93]. This improvement was attributed to reduced inflammatory activation of endothelial cells and the recruitment of leukocytes, exacerbating cardiac damage [93]. Likewise, the delivery of longevity-associated variant (LAV) of bactericidal/permeability-increasing fold-containing family B member 4 (BPIFB4) attenuates diabetes-induced cardiac dysfunction through anti-inflammatory action [94]. LAV-BFIB4 allele was demonstrated to be highly prevalent in long-living individuals with higher circulating BPIB4 levels with increased eNOS and mononuclear cells [95]. A recent study looking at liver targeted (thyroxine-binding globulin) administration AAV9-LAV-BFIB4 demonstrated improvement in cardiac function and remodelling in diabetic mice [94]. These beneficial effects have been attributed to increasing circulating BFIB4 that increases stromal-derived factor-1 (SDF)-1 release, that in turn, activate CXC chemokine receptor type 4 (CXCR-4) [94]. Despite the positive outcomes demonstrated by these studies, it is important to note that some of these studies are still in the early stages and future work looking into the use of AAV gene therapies to limit ROS production (e.g., knockdown of Nox subunits) or increase antioxidant enzymes (e.g., increase in SOD) in the diabetic heart are still required.

### Myofilament/contractile function targeted AAVs

Cardiac manipulation to increase myofilament cross-bridge binding and cycling was investigated to improve cardiac function by increasing naturally occurring nucleotide, 2-deoxyadenosine triphosphate (dATP) [96]. Production of dATP is mainly maintained by ribonucleotide reductase (RNR), by converting adenosine diphosphate (ADP) to deoxy-adenosine diphosphate (dADP), leading to the final production of dATP. Targeting RNR with the administration of Rrm-1 and Rrm-2 genes (which encodes RNR) using an AAV approach improved LV systolic and diastolic function in rodent and porcine models of myocardial infarction [96,97]. This cardioprotective effect was further confirmed in Duchenne Muscular Dystrophy mice, where similar cardiac improvements were observed [98]. Similarly, inhibition of β-adrenergic receptor kinase (βARK), which is increased in heart failure, displays beneficial effects in the heart. Indeed, administration of AAV6-βARKct (the peptide responsible for βARK inhibition) showed improvements in LV haemodynamic and contractile function were evident in a pig model of myocardial infarction [99]. This was also observed in cardiomyocytes obtained from failing human hearts, where there is improved contractile function and increased adenylyl cyclase activity in response to AAV6-βARKct, indicating improved β-adrenergic receptor activity, a major regulator of cardiac contractility [100]. Further, studies investigating the toxicity and safety of AAV6-βARKct in sheep reported no toxic effects on major organ function, with robust cardiac gene expression [101], suggesting that this treatment may be close to clinical translation.

### AAVs targeting post-translational modification mechanisms

Post-translational modification, a chemical process that occurs following protein biosynthesis, is an important component to cell signalling and function including the heart. Some of these modifications include phosphorylation, acetylation, nitrosylation, alkylation and glycosylation. One that is particularly attractive to target in the context of diabetes and its complications is O-GlcNAcylation, a modification that is glucose-driven and has been implicated in the development of diabetic cardiomyopathy [13]. Two enzymes regulate this post-translational modification: O-GlcNAc transferase (OGT), which facilitates the addition of the O-GlcNAc sugar moiety to Ser and Thr residues, and O-GlcNAc-ase (OGA), which facilitates its removal [102]. Our laboratory has shown that administration of rAAV6 carrying a human isoform of OGA attenuated cardiac dysfunction and remodelling in mice with established diabetic cardiomyopathy [103,104]. In contrast, administration of rAAV6 carrying a human isoform of OGT induces cardiac dysfunction and remodelling in wild-type non-diabetic mice [103,104] This suggests that regulation of this particular post-translational modification is a critical regulator of the structural and functional phenotype of the heart, and might be key in the setting of cardiomyopathy, both with and without diabetes.

### AAVs targeting cardiac apoptosis

Cardiac apoptosis is a common feature of the diabetic heart and end-stage heart failure in both clinical and preclinical contexts [7]. The pro-viral integration site for Moloney murine leukaemia virus (PIM-1) has been identified as a promotor of cardiomyocyte survival in response to cell stress [105]. Increased cardiac expression of PIM-1 via AAV9 delivery increased proliferation of cardiac progenitor cells and improved cardiac contractility, with evidence of increased SERCA2a activity [106]. Likewise, the lipid kinase PI3K(p110α) responsible for membrane trafficking, cell growth and cell survival, has also been shown to be cardioprotective in multiple cardiac disorders. Administration of rAAV6-caPI3K gene therapy was demonstrated to diminish cardiac dysfunction following acute pressure-overload hypertrophy [107]. In a chronic disease such as diabetes, we have shown that administration of rAAV6-caPI3K gene therapy to mice with type-1 diabetes-induced diastolic dysfunction, attenuated cardiac dysfunction and oxidative stress [108]. Equally, in a preclinical model of type-2 diabetes we have also demonstrated that rAAV6-caPI3K administration delivers similar cardiac improvements [109].

### AAVs targeting micro RNAs (miRNA)

Micro RNAs (miRNA) are small, non-coding RNAs that function via base-pairing with complementary sequences and messenger RNA molecules. These can regulate multiple different genes at the post-transcriptional level, in both healthy and disease settings [110]. Indeed, diabetes-induced oxidative stress, hypertrophy and cardiac fibrosis have been shown to be associated with changes in several miRNAs, including miRNA-1, miRNA-133a, miRNA-373, miRNA-378, miRNA-23b, miRNA-181 and miRNA-195 [110]. Thus, there is an increased interest in targeting miRNA for the treatment of diabetic cardiomyopathy. One mode of delivery that has been widely used for cardiac miRNA studies is the use of AAV as a vehicle, due to its versatility and capability to transduce cardiac tissue. For instance restoration of miR-1 using AAV9 improved fractional shortening, attenuated cardiac remodelling and improved Ca^2+^ cycling in rats with aortic banding [111]. In the setting of diabetic cardiomyopathy, delivery of cardiac-targeted AAV9 carrying miRNA-21 has been shown to attenuate diabetes-induced cardiomyocyte hypertrophy and diastolic dysfunction, via gelsolin inhibition, an important cardiac transcription cofactor [112]. Similarly, administration of rAAV9-miRNA-30c has also been demonstrated to limit diabetes-induced cardiac dysfunction and remodelling in T2D *db/db* mice [113]. This has been attributed to an increase in peroxisome proliferator-activated receptor (PPAR)-α transcriptional activity that modulates oxidative stress, lipid accumulation, ATP production and apoptosis in the diabetic heart [113]. In contrast with the cardioprotective effects demonstrated for the above miRNAs, an increase in miRNA-320 has been shown to be detrimental in the setting of diabetic cardiomyopathy [114]. Delivery of rAAV-miRNA-320 exacerbates cardiac remodelling and dysfunction in T2D mice, whereas inhibition of miRNA-320 attenuated these cardiac parameters [114]. These studies indicate that regulation of miRNA in the diabetic heart is complex and will benefit from further investigation.

## Clinical translation of AAV-based therapeutics

**Figure 3:**
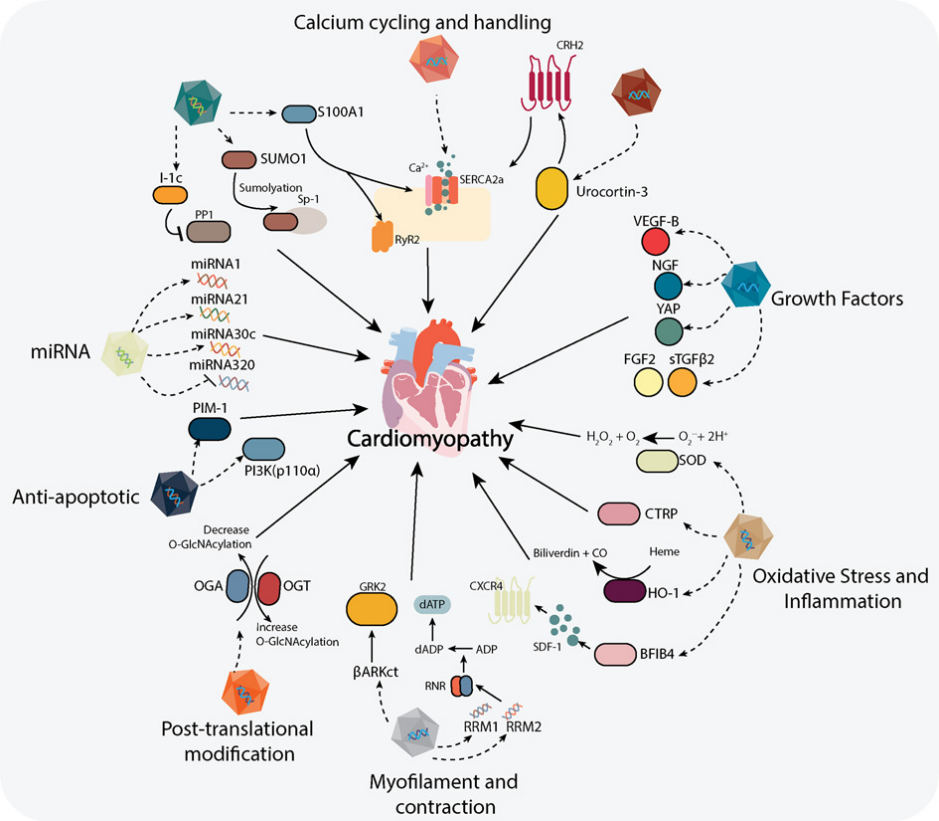
Outline of reported AAV-based potential therapeutics for diabetic cardiomyopathy Current AAV gene therapies reported to date to target multiple signalling pathways and molecules in cardiac tissue proposed for the treatment of diabetic cardiomyopathy. ADP, adenosine diphosphate; βARKct; β-adrenergic receptor kinase 1 (carboxy terminus); BPIFB4, bactericidal/permeability-increasing fold-containing family B member 4; CRH_2_, corticotrophin-releasing hormone receptor-2; CTRP, C1q/tumour necrosis factor-related protein; dADP, deoxyadenosine diphosphate; dATP, 2-deoxyadenosine triphosphate; FGF, fibroblast growth factor-2; GRK2; G protein-coupled receptor kinase-2; HO-1, heme oxygenase-1; I-1c, constitutively active inhibitor-1; miRNA, micro RNA; NGF, nerve growth factor; OGA, O-GlcNAc-ase; OGT, O-GlcNAc-transferase; PI3K(p110α), phosphoinositide 3- kinase (p110α); PIM-1, pro-viral integration site for Moloney murine leukaemia virus; PP1, protein phosphatase-1; RNR, ribonucleotide reductase; Ryr2, ryanodine receptor-2; S100A1, S100 calcium-binding protein A1; SDF-1, stromal-derived factor-1; SOD, superoxide dismutase; Sp-1; specificity protein-1; sTGFβ2, soluble transforming growth factor-β2; SUMO-1, small ubiquitin-related modifier-1; SERCA2a, sarcoplasmic/endoplasmic reticulum ATPase-2; VEGF-B, vascular endothelial growth factor-B; YAP, yes-associated protein-1 (see text for references).

The Calcium Upregulation by Percutaneous Administration of Gene Therapy in Patients with Cardiac Disease (CUPID) trial was the first human study that investigated the use of AAV for heart failure by targeting impaired Ca^2+^ handling utilising SERCA2a-AAV gene therapy [115]. This treatment aimed to treat heart failure through an increase in SERCA2a protein (responsible for regulating calcium reuptake following contraction) [40]. Results from both Phase 1 and Phase 2 clinical trials were promising, demonstrating no or few side effects in the small patient cohort [50,116]. However, the results of the Phase 2b clinical trial were neutral, as the administration of SERCA2a-AAV did not achieve improvement in primary end point in both recurring (i.e. admission to hospital due to heart failure) and terminal events (i.e. death) [117]. However, a clinical trial using adenylyl cyclase-6 gene delivery in heart failure patients demonstrated favourable outcomes in terms of improving LV ejection fraction and LV-dP/dt [118]. The improvement has been attributed to an increase in SERCA2a, Akt and phospholamban following treatment [119,120]. Currently, a Phase 3 clinical trial is being planned and reassessed for commencement to investigate the effects of intracoronary administration of Ad5 CMV hAC6 (RT-100) or placebo in 536 HFrEF (Heart Failure with reduced Ejection Fraction) patients (FLOURISH Trial) ([121]- Clinical Trials.gov NCT03360448). More recently I-1c-AAV gene therapy (a protein phosphatase-1 inhibitor), which demonstrated success in preclinical studies, has progressed to a Phase 1 clinical trial, to investigate the safety profile of NAN-101 (BNP116.sc-CMVi1c) in patients with heart failure (Clinical Trials.gov NCT04179643, start date 20 November 2019). Thus, these studies highlight that therapeutic gene approaches are in the pipeline for the treatment of heart-related diseases (Figure 3), which have proven to offer clear promise at least in the preclinical stage.

## Challenges and hurdles posed by AAV-mediated gene delivery approaches

Over the last few decades, the field of gene therapy has advanced significantly in terms of vector design and biology. Although the findings of the first gene therapy for heart failure was negative [117], leading gene technology experts have pointed out that further optimisation is still required to translate AAV use from bench to bedside [122]. Findings from the CUPID trial highlighted poor uptake of the AAV vector into the hearts of patients [117]. Other factors, including the presence of neutralising antibodies, have been considered a hurdle, where even at low concentration can inactivate AAV activity and block transduction to target tissue [123]. However, new strategies have been developed to tackle this limitation. Formation of synthetic AAVs through structural guided evolution and generation of chimeric AAV via fusion of wild-type AAV have been some of the few technologies that have in the developmental pipeline [124,125]. Recent findings have also highlighted the potential for toxic effects of AAVs in non-human primates [126]. Administration of a high dose AAV (2 × 10^14^ vg/kg) demonstrated severe hepatotoxicity, with side effects evident in the dorsal root ganglia [126]. These findings have been debated in the field of gene therapy and argued to be a variant-specific effect; in this case AAVhu68 (an AAV9 variant) was used, a variant that has not been used in the clinic [127]. Moreover, the dose that was administered was very high compared with that which is currently being used in the clinic (e.g. 1.5 × 10^11^ vg based on the current, clinically approved therapy for retinal dystrophy, voretigene neparvovec-rzyl). Despite these findings, a gene therapy product, Gendicine (recombinant human p53 adenoviral), to treat squamous cell carcinoma, was approved for clinical use in China over a decade ago [128]. There have been no adverse events reported and this therapy has continued to demonstrate an exemplary safety profile in the clinic [128].

The advantage of gene therapy over conventional pharmacological therapies is their potential to last for a prolonged period, if not a lifetime [51]. As these therapies only need to be administered once, or up to a handful of times, the cost per administration is likely to be eye-watering, as pharmaceutical companies look to recoup the funds invested in research and development (ranging from US$500,000 to $2 million per course of treatment) [129]. The high price-tag has been associated with low commercialisation of this therapy in the marketplace, as most of the currently approved therapies target rare monogenic genetic defects that only occur in a small number of people. For example, Zolgensma, a recently approved gene therapy treatment for spinal muscular atrophy (a disorder that affects 1 in 10,000 live births www.rarediseases.org) costs US$2 million per treatment course [130]. Although not unique to AAV gene therapy, new therapies had led pharmaceutical companies to devise innovative schemes to cover the costs of gene therapy, including pay for performance; where payment is only made when maintenance or improvement of health is maintained over a period of time. Additionally, subscription-based or money-back guarantees/rebate model is also another option that had been recently adopted by health care providers throughout the world [129]. Nonetheless, like all technologies, the cost of gene therapy will eventually reduce as research and development costs decrease over time, increasing the feasibility of AAV gene delivery as a therapy from an economic perspective. Finally, the increasing evidence and research in the treatment of diabetic cardiomyopathy may also influence the need for such targeted interventions.

## AAV-mediated gene delivery therapeutic approaches – are we there yet?

The use of gene therapy has emerged to be one of the most versatile approaches in the biomedical field. It can be used to manipulate targets of interest by either up-regulating or down-regulating the activity of the target gene or protein in a tissue-specific manner, which is desirable to dissect specific pathways in different disease pathologies. Likewise, the use of AAVs in the clinic is a viable therapeutic strategy to deliver beneficial genetic materials, in place or in conjunction with pharmacological interventions. In addition, the advancement of gene therapeutics is highlighted by the increase in clinical trials (364 clinical trials based on literature search for ‘cardiovascular diseases’ and ‘gene therapy’ in www.clinicaltrials.gov as of June 2021). At time of writing, there were 3 AAV gene therapies (2 available in the clinic and 1 (Glybera) withdrawn due to expensive regulatory costs and low revenues) that have been approved for clinical use (Table 2). Indeed AAV gene technology has also been investigated as a COVID-19 vaccine and has been shown to be stable at room temperature with a favourable safety profile compared to first-generation vaccine [131]. With this resurgence and our ever-increasing knowledge in the field, it will lead to reduced research and development costs, coupled with innovative ways to recoup costs and increased level of industry-sponsored funding in the academic sector can further expand the gene therapy developmental pipeline. Moreover, significant investment is being made to optimise the protocols required for manufacturing vector stocks for clinical use, such that production can be scalable and yield a vector at the highest possible titre [132].

**Table 2:** Current list of approved AAV gene therapy products for clinical use

Therapeutic name	Year of approval	Approving agency	Indication	Type of therapy	Vector	Dose	Route of administration	Manufacturer
Glybera^*^ (alipogene tiparvovec)	2012	EMA	Lipoprotein lipase deficiency	AAV gene therapy	AAV1-LPL	1 × 10^12^ vg/kg body weight	Intramuscular injection	UniQure (Amsterdam, Netherlands)
**Luxturna** (voretigene neparvovec-rzyl)	2017/2018	FDA/EMA	Retinal dystrophy (biallelic RPE65 mutation)	AAV gene therapy	AAV2-RPE65	1.5 × 10^11^ vg/eye	Subretinal injection	Spark Therapeutics, Inc (Philadelphia, Pennsylvania, U.S.A.)
**Zolgensma** (onasemnogene aberparvovec xioi)	2019	FDA	Spinal muscular atrophy	AAV gene therapy	AAV9- SMN1	1.1 × 10^14^ vg/kg body weight	Intravenous infusion	AveXis Inc (Chicago, Illinois, U.S.A.)

EMA, European Marketing Authorization; FDA, Food and Drug Administration.

* prohibitive cost of regulatory body precluding commercial viability.

As discussed here, AAV gene therapy for diabetic cardiomyopathy in the clinic is now getting closer to becoming a reality. This can be attributed to a better understanding of the delivery vectors and promoters, their modes of delivery and improved knowledge of the molecular targets. However, challenges to achieve efficient gene transduction remain as the limiting factor for the translation of preclinical models to the clinic. In addition, the decision regarding which molecular pathways are targeted is also critical to achieving successful gene therapy transduction and outcome. Nevertheless, the capacity for gene therapy to cure human disease is now an established reality in multiple diseases. It is now only a matter of time before one will be available for the treatment of diabetic cardiomyopathy, for which there is currently no cure.

## Open Access

Open access for this article was enabled by the participation of University of Monash in an all-inclusive *Read & Publish* pilot with Portland Press and the Biochemical Society under a transformative agreement with CAUL.

## Abbreviations

αMHC: α-myosin heavy chain
βARK: β-adrenergic receptor kinase
AAV: Adeno-associated viral vector
ACEi: Angiotensin converting-enzyme inhibitors
BPIFB4: Bactericidal/permeability-increasing fold-containing family B member 4
CMV: cytomegalovirus
CRH2: corticotropin-releasing hormone receptor 2
CTGF: connective tissue growth factor
CTRP: C1q/tumour necrosis factor-related protein
CXCR4: CXC chemokine receptor type 4 (CXCR4)
dATP: 2-deoxyadenosine triphosphate
DPP4: dipeptidyl peptidase-4
eNOS: endothelial nitric oxide synthase
FGF-2: fibroblast growth factor-2
Foxo: Forkhead box-O-transcription factor
GLP-1: glucagon-like peptide-1
HO-1: heme oxygenase-1
LAV: longevity-associated variant
LV: left ventricle
MLC: myosin light chain
NF-κB: nuclear-factor-κB
NGF: nerve growth factor
Nox: NADPH Oxidase
O-GlcNAc: O-linked N-acetylglucosamine
OGA: O-GlcNAc-ase
OGT: O-GlcNAc transferase
PI3K: phosphoinositide-3 kinase
PIM-1: Pro-viral integration site for Moloney murine leukaemia virus
Pln: phospholamban
rAAV: recombinant AAV
RNR: ribonucleotide reductase
ROS: reactive oxygen species
Ryr2: ryanodine receptor-2
S100A1: S100 calcium-binding protein A1
SDF-1: stromal derived factor-1
Ser: serine
SERCA2a: sarcoplasmic/endoplasmic reticulum Ca^2+^-ATPase
SGLT-2: sodium-glucose co-transporter-2
SOD: superoxide dismutase
Sp-1: specificity protein-1
SUMO-1: small ubiquitin-related modifier-1
TGF-β: transforming growth factor-β
Thr: threonine
TNF-α: tumour necrosis factor-α
TZD: thiazolidinedione
VEGF: vascular endothelial growth factor
YAP: Yes-associated protein
